# Characterization of a Novel Heterochromatin Protein 1 Homolog “*HP1c*” in the Silkworm, *Bombyx mori*

**DOI:** 10.3390/insects13070631

**Published:** 2022-07-14

**Authors:** Masato Hino, Tsuneyuki Tatsuke, Akihiro Morio, Hiroaki Mon, Jae Man Lee, Akitsu Masuda, Kohei Kakino, Yoshino Tonooka, Takahiro Kusakabe

**Affiliations:** 1 Laboratory of Sanitary Entomology, Graduate School of Bioresource and Bioenvironmental Sciences, Kyushu University, Motooka 744, Nishi-ku, Fukuoka 819-0395, Japan; m.hino.018@agr.kyushu-u.ac.jp; 2 Laboratory of Insect Genome Science, Graduate School of Bioresource and Bioenvironmental Sciences, Kyushu University, Motooka 744, Nishi-ku, Fukuoka 819-0395, Japan; tatsuket438@affrc.go.jp (T.T.); morio.akihiro.ad@daiichisankyo.co.jp (A.M.); mhiro@agr.kyushu-u.ac.jp (H.M.); a.masuda@agr.kyushu-u.ac.jp (A.M.); k.kakino@agr.kyushu-u.ac.jp (K.K.); tonookay@sc.sumitomo-chem.co.jp (Y.T.); 3 Laboratory of Creative Science for Insect Industries, Graduate School of Bioresource and Bioenvironmental Sciences, Kyushu University, Motooka 744, Nishi-ku, Fukuoka 819-0395, Japan; jaemanle@agr.kyushu-u.ac.jp

**Keywords:** *Bombyx mori*, heterochromatin protein 1, knockdown, intracellular localization, novel gene, transcriptional repression

## Abstract

**Simple Summary:**

Heterochromatin protein 1 (HP1) plays a major role in the formation and maintenance of heterochromatin and in the regulation of gene expression. Five *HP1* genes have been found in *Drosophila melanogaster* and three *HP1* genes in *Homo sapiens*, while in *Bombyx mori*, two *HP1* genes (*BmHP1a* and *BmHP1b*) have been reported. In the present study, we analyzed the function of the novel *Bombyx mori HP1* gene (*BmHP1c*), the third *HP1* gene in silkworm. BmHP1c has different characteristics from BmHP1a and BmHP1b in terms of transcriptional repression activity, dimer formation, subcellular localization, and effects of RNAi on cell cycle progression. These findings indicate that BmHP1c plays a different role than BmHP1a and BmHP1b.

**Abstract:**

Heterochromatin protein 1 plays an important role in chromatin structure and gene expression regulation. Three *HP1* genes have been found in *Homo sapiens*, and five *HP1* genes have been reported in *Drosophila melanogaster*. On the other hand, in *Bombyx mori*, only two *HP1* genes, *BmHP1a* and *BmHP1b*, were reported. In this research, we have reported the molecular and functional characterization of a novel *Bombyx mori HP1* gene (*BmHP1c*), which had stronger transcriptional repression activity than BmHP1a. BmHP1a and BmHP1b is reported to form homo- and heterodimers, but in co-immunoprecipitation experiments, no homo- or hetero-dimer formation of BmHP1c with the other silkworm HP1s is detected. The intracellular localization of BmHP1c is not only in the nucleus but also in the cytoplasm like mammalian HP1*γ*. In contrast to human HP1*a* and b, all three BmHP1s were localized preferentially in the regions poorly stained with DAPI. Interestingly, the double knockdown of *BmHP1a* and *b*, but not *BmHP1c* with *a* or *b*, arrested the cell cycle at the G2/M phase. These results suggest that BmHP1c is not essential for cell progression and plays a different role than BmHP1a and BmHP1b.

## 1. Introduction

Eukaryotic chromatin has two states, transcriptionally repressive heterochromatin and active euchromatin. Generally, in the euchromatic region, the initiation of DNA replication is earlier than in the heterochromatic region [1,2]. In mammalian chromosomes, H3K4me1/2/3 and H3K36me3 are known as typical histone modifications for active regions [3,4]. On the other hand, the heterochromatic regions are hypoacetylated, and H3K9me2/3 and H3K27me2/3 are known as histone modifications for inactive regions [3,4]. Among these, the methylation of histone H3K9 mediated by *SUV39H* provides the scaffold for the binding of heterochromatin protein 1 (HP1) and plays a significant role in the programmed gene expression [5]. HP1 plays a predominant role in the organization and maintenance of heterochromatin, where the transcription is suppressed, especially in the regions enriched in transposable elements.

HP1 has two domains, chromodomain (CD) and chromo shadow domain (CSD). CD recognizes the methylation of histone H3K9 and binds to histone H3K9me [6]. CD domain is also found in *SUV39H* family proteins and polycomb (*Pc*) proteins. CSD is a domain found only in HP1 and contributes to the dimerization among HP1s and interaction with HP1-related proteins [7,8,9,10].

Functional and structural studies on HP1s have been actively conducted in human, mouse, and fruit fly, which have monocentric chromosomes. In *Drosophila* HP1s, DmHP1*a* is predominantly located in the heterochromatin region [11,12] and plays two roles in the up- and downregulation of transcription [12,13,14]. On the other hand, DmHP1b is present in both heterochromatin and euchromatin [11] and has been shown to upregulate transcription in general [15], but there are also a few results showing that DmHP1b represses transcription [12,14]. As for DmHP1c, it differs significantly from the general definition of HP1, is abundant in the euchromatin region [11,12], and activates transcription [12,14]. In the knockdown experiments of *DmHP1a*, *b*, and *c*, respectively, using *Drosophila* S2 cells, no regularity or striking similarity has been found between genes up- or down-regulated by knockdown [12,16,17]. In addition, the genes whose expression is altered by inhibition of *DmHP1a* differ among cell types [13,16,17], indicating that the function of HP1s is still poorly understood. In addition to these, *Drosophila* has two other *HP1* genes, *DmHP1d* and *DmHP1e*, that are expressed in a tissue-specific manner.

Very little research has been done on *HP1s* in the silkworm *Bombyx mori*, which has a unique holocentric chromosome, and there are many more unknowns than in the typical model organisms described above. The silkworm genome is reported to encode two functional *HP1s*: *BmHP1a* and *BmHP1b* [18]. BmHP1a and BmHP1b form homo- or hetero-dimer and interact with *BmSu(var)3–9*, a *SUV39H* family protein [18]. From the results of ChIP-seq analyses, however, the accumulation of heterochromatin-specific histone tail modifications was not observed in the binding sites of BmHP1a, which indicates its role in transcriptional activation [19]. In addition to two *BmHP1s*, the silkworm genome encodes *BmCdp1* with two chromodomains, and Shoji et al. (2013) [20] showed by ChIP analysis that it binds to eight regions of the silkworm chromosome, including the upstream region of the putative gene BGIBMGA007060. When *BmCdp1* was depleted or knocked down in BmN4 cells or silkworm embryos, respectively, there was no obvious effect on the expression of BGIBMGA007060, and no phenotypic abnormality was observed.

In this study, we have identified *BmHP1c* as a new HP1 gene and analyzed its function. BmHP1c had a transcriptional repression activity equivalent to that of BmHP1b, which is stronger than that of BmHP1a. Interestingly, BmHP1c showed a unique localization in the cultured silkworm cells. BmHP1c existed in both the nucleus and the cytoplasm, although BmHP1a and BmHP1b localized in the nucleus. In addition to this difference, Mitsunobu et al. [18] reported that BmHP1a and BmHP1b forms homo- and heterodimers, whereas the formation of BmHP1c homo- and heterodimers with BmHP1s was not observed. It is necessary to evaluate the relationship with proteins that interact with other HP1s and to verify the function of BmHP1c in more detail.

## 2. Materials and Methods

### 2.1. Cell Culture

BmN4 (kindly provided by Dr. Chisa Aoki, Kyushu University Graduate School, Fukuoka, Japan) and BmN4-SID1 were cultured with IPL41 insect medium containing 10% fetal bovine serum (Gibco, Grand Island, NY, USA) at 27 °C.

### 2.2. Accession Numbers

The database accession numbers of proteins are as follows: *B. mori Cdp1* (NP_001272895.1); *B. mori* HP1a (NP_001040539.1), b (NP_001159616.1), c (XP_004923534.2); *D. melanogaster* HP1a (NP_476755.1), b (NP_001162713.1), c (NP_651093.1), *d* (NP_536794.1) and *e* (NP_649878.1); *H. sapiens* HP1*α* (NP_001120793.1), *β* (NP_001120700.1) and *γ* (NP_009207.2); *M. musculus* HP1*α* (NP_001070257.1), *β* (NP_031648.1) and *γ* (NP_001341931.1); *S. pombe Swi6* (NP_593449.1).

### 2.3. Domain Search of HP1

Amino acid sequences of various organisms were retrieved from the NCBI database. The database accession numbers are listed in Section 2.2. DoMosaics was used for the domain search. Chromo and Chromo_shadow represent the chromatin organization modifier domain and the chromo shadow domain in the Pfam database, respectively (Chromo accession number: PF00385, Chromo_shadow accession number: PF01393).

### 2.4. Phylogenetic Tree

Amino acid sequences of various organisms were retrieved from the NCBI database. The database accession numbers are listed in Section 2.2. A phylogenetic tree was created using ClustalW and drawn using JalView software in which a neighbor-joining method was used to calculate. The scores written in the phylogenetic tree represent the distances. The chroma shadow domain region was identified using a website “MOTIF: Searching Protein Sequence Motifs—GenomeNet” (https://www.genome.jp/tools/motif/, accessed on 28 June 2022) and Pfam database.

### 2.5. Transcription Repression Assay

The *BmHP1s* coding regions in pENTR were inserted into the pIE2-HA-Gal4DBD-DEST vector by using of Gateway LR reaction (Life Technologies, Carlsbad, CA, USA). The pIE2-HA- Gal4DBD-DEST vector is an expression vector that fuses a hemagglutinin (HA) tag and DNA binding domain of Gal4 to the N terminus of the genes of interest [18]. Each vector was transfected to BmN4 cells with the Luc2P reporter plasmid, which contains five GAL4DBD binding sites (UAS) upstream of the Bmhsp promoter and *Luc2P* gene [18]. Five days after transfection, the cells were collected, washed with 1 × PBS, and lysed with 200 µL of lysis buffer (25 mM Tris-phosphate pH 7.8, 2 mM DTT, 2 mM Trans-1, 2-diaminocyclohexane-N,N,N′,N′-tetraacetic acid monohydrate, 10% glycerol, 1% Triton X-100). The lysates were centrifuged at 14,000 rpm for 1 min, and the supernatants were used for measuring the luciferase activity. After mixing the supernatant and the luciferase substrate, the luciferase activities were measured by ARVO (Perkin Elmer, Boston, MA, USA).

The principle is explained below. The Luc2P reporter plasmid contains the *Luc2P* gene at downstream of the Bmhsp promoter and five GAL4DBD binding sites (UAS) upstream of the Bmhsp promoter. The GAL4DBD-fused protein expressed from the GAL4DBD vector binds to the upstream of the Bmhsp promoter of the Luc2P reporter plasmid. If GAL4DBD-BmHP1 has transcriptional repressive activity, the expression of Luc2P should be reduced and the value of luciferase activity should be lower compared to the negative control (GAL4DBD-EGFP).

### 2.6. Co-Immunoprecipitation Assay

The *BmHP1s* coding regions in entry vectors were transferred into pIE2-HA-DEST vector or pIE2-Flag-DEST vector by using Gateway LR reaction (Life Technologies, Carlsbad, CA, USA). The pIE2-HA-DEST vector is an expression vector that fuses a hemagglutinin (HA) tag to the N terminus of the genes of interest, and the pIE2-Flag-DEST vector is an expression vector that fuses a Flag tag to the N terminus of the genes of interest [18]. These two types of vectors were mixed and transfected into BmN4 cells using six-well plates and Avalanche^®^-Everyday Transfection Reagent (APRO Science). Five days after transfection, the cells were collected, washed with 1 × PBS, and lysed with 200 µL of lysis buffer (10 mM Tris-HCl pH 7.5, 150 mM NaCl, 5 mM EDTA, 1% Nonidet P-40, protease inhibitor cocktail Complete). After incubation for 20 min in ice, the cells were homogenized using Handy Sonic model UR-20P (Tomy Seiko, Tokyo, Japan) at power level 6. After centrifugation at 14,000 rpm for 30 min at 4 °C, the input samples were prepared by mixing the 20 µL supernatants with an equal volume of 2x SDS sample buffer (100 mM Tris-HCl pH6.8, 200 mM DTT, 4% SDS, 0.02% bromophenol blue, 20% glycerol) and boiling at 95 °C for 5 min. The remaining supernatants were incubated with anti-Flag (or -HA) monoclonal antibody-coupled G protein beads (GE Healthcare Life Sciences) at 4 °C overnight. The beads were precipitated by centrifugation, and 20 µL of this supernatant was mixed with an equal volume of 2x SDS sample buffer and boiled at 95 °C for 5 min to prepare unbound samples. The beads were washed three times with the lysis buffer and boiled in 50 µL SDS sample buffer (bound samples). IP samples were subjected to sodium dodecyl sulfate-polyacrylamide gel electrophoresis (SDS-PAGE) and western blotting.

### 2.7. Western Blotting

Western blotting was performed using the method described in Hino et al. [21]. Here is a brief description. Protein samples obtained by co-immunoprecipitation were subjected to 12% or 10% SDS-PAGE and further transferred to polyvinylidene difluoride (PVDF) membrane. After blocking in TBST buffer (20 mM Tris-HCl pH 7.6; 500 mM NaCl; 0.1% *w*/*v* Tween-20) with 3% *w*/*v* skim milk (Wako, Japan), the membrane transferred from 10% gel was subsequently incubated with anti-Flag antibody (1:1000 *v*/*v*; Sigma, no. F3165), and the other membrane transferred from 12% gel was subsequently incubated with anti-HA antibody (1:1000 *v*/*v*; Sigma, no. H9658) at 4 °C overnight. The next day, the membranes were incubated at 37 °C. After a three-time TBST buffer wash, the membranes were incubated with HRP-labeled anti-mouse IgG (1:4000, Sigma, no. A9044) at 37 °C for 2 h. The membranes were then washed five times, and the HRP signals were visualized using Super Signal West Pico Chemiluminescent Substrate (Thermo Fisher Scientific, Waltham, MA, USA).

### 2.8. Semi-Quantitative RT-PCR

Total RNA was extracted using ISOGEN. Then, reverse transcription was performed using ReverTra Ace to prepare cDNA. PCR was performed using gene-specific primers (Table 1) to confirm the gene expression in the different tissues and the efficiencies of gene knockdown.

### 2.9. Fluorescence Microscopic Observation

In order to analyze the subcellular localization of BmHP1c in cultured silkworm cells, the cells expressing *mClover3* fused BmHP1c were observed using a confocal laser microscope. pIE2-*mClover3*-BmHP1c vector was made by Gateway LR reaction (Life Technologies, Carlsbad, CA, USA) between BmHP1c -pENTR11 and pIE2-*mClover3*-DEST. The pIE2-*mClover3*-DEST vector is an expression vector that fuses the fluorescent protein *mClover3* to the N-terminus of the genes of interest. Three days after the transfection of pIE2-*mClover3*-BmHP1c, the cells were seeded on a cover glass whose surface was coated with Poly-L-Lysine in a six-well plate and incubated for one day. Thereafter, the cells were fixed with 4% paraformaldehyde in 1 × PBS buffer for 15 min at room temperature. After washing three times with 1 × PBS, it was mounted on a microscope slide using ProLong™ Diamond Antifade Mountant with DAPI (Thermo Fisher Scientific, Waltham, MA, UAS), and it was incubated overnight at room temperature in the dark. Finally, the cells were observed under a confocal laser microscope (TCS SP8, Leica, Wetzlar, Germany). This microscope was provided by the Center for Advanced Instrumental and Educational Supports, Faculty of Agriculture, Kyushu University. Leica Application Suite X (LAS X) was used for the analysis.

### 2.10. RNA Interference

Knockdown experiments were performed to confirm the effects on the cell cycle. In this experiment, BmN4-SID1 cells, which can induce gene knockdown simply by adding dsRNA to the medium, were used [22]. To prepare dsRNA, the target gene region was amplified with T7 promoter sequence added primers (5′- TAATACGACTCACTATAGGGTTTGTACAAAAAAGCAGGCT-3′ and 5′-TAATACGACTCACTATAGGGACTTTGTACAAGAAAGCTGG-3′) using the entry vector containing each *BmHP1* as a template, and transcription was performed using the amplified PCR fragments and T7 RNA polymerase. RNA interference was induced by adding dsRNA to the medium.

### 2.11. Flow Cytometry

Gene knockdown cells were fixed with 70% ethanol. After digesting the intracellular RNA with RNase A, the cells were stained with propidium iodide, and the cell cycle was measured using a Guava PCA-96 Flow Cytometer (Millipore, Burlington, MA, USA).

## 3. Results

### 3.1. BmHP1c Was Identified as a Novel HP1 Gene

**Figure 1:**
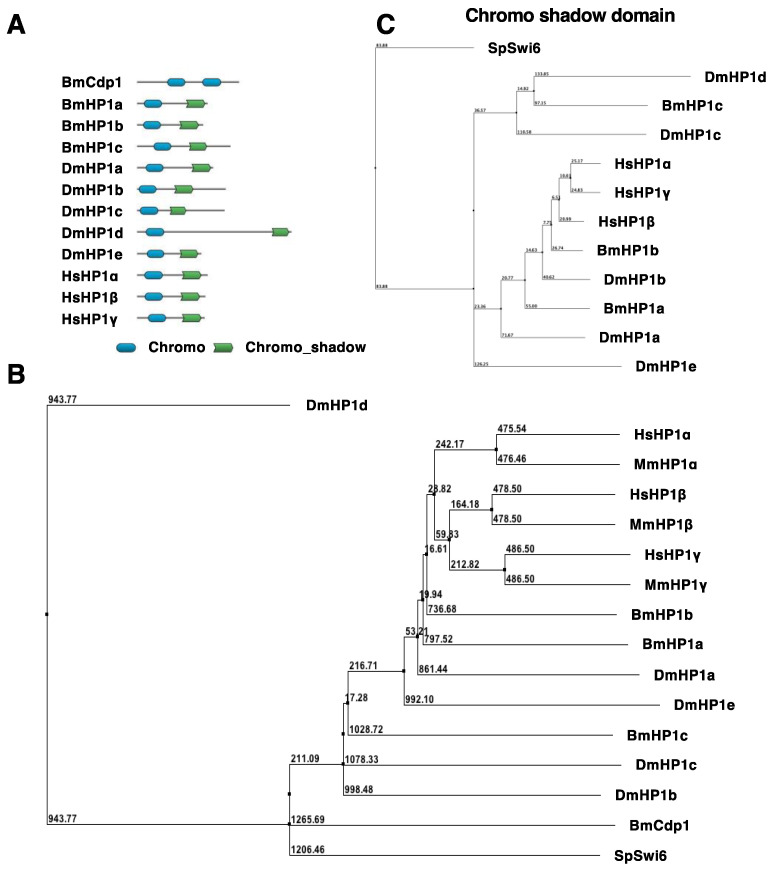
(**A**) Domain search of HP1 of various organisms (*Bombyx mori*, *Drosophila melanogaster*, *Homo sapiens*). (**B**) Phylogenetic tree of HP1 of various organisms (*Bombyx mori*, *Drosophila melanogaster*, *Homo sapiens*, *Mus musculus*, *Schizosaccharomyces pombe*). (**C**) Phylogenetic tree of chromo shadow domain which HP1s of various organisms have (*Bombyx mori*, *Drosophila melanogaster*, *Homo sapiens, Schizosaccharomyces pombe*).

In silkworm, two functional *HP1* genes, *BmHP1a* and *BmHP1b*, have been reported [18]. To reconfirm the composition of the *HP1* gene family in the silkworm, we analyzed the de novo assembled transcriptome of BmN4 cells, which was constructed by the Insect Genome Science Laboratory. As a result, *BmHP1c* was identified as an additional silkworm *HP1* gene. When we checked the NCBI database, this gene was registered as LOC101738835 (Gene symbol) in NCBI. BmHP1c has one chromodomain and one chromo shadow domain like BmHP1a and BmHP1b (Figure 1A). *BmHP1a*, *BmHP1b*, and *BmHP1c* encode 191 (MW = 21.9 kDa), 177 (MW = 20.4 kDa) and 262 amino acid residues (MW = 29.5 kDa), respectively. BmHP1c is the largest protein and has the longer N- and C-terminus sequences than BmHP1a and BmHP1b. In the phylogenetic tree analysis, BmHP1a and BmHP1b were located in the same cluster, but BmHP1c was located out of the cluster (Figure 1B). In addition, in the phylogenetic tree of CSDs, BmHP1a and BmHP1b belong to the same cluster, while BmHP1c belongs to a different cluster (Figure 1C). These results suggested that BmHP1c may behave differently from BmHP1a and BmHP1b, so we investigated whether BmHP1c has the same transcriptional repression activity as the other BmHP1s.

### 3.2. BmHP1c Has Transcriptional Repression Activity

**Figure 2:**
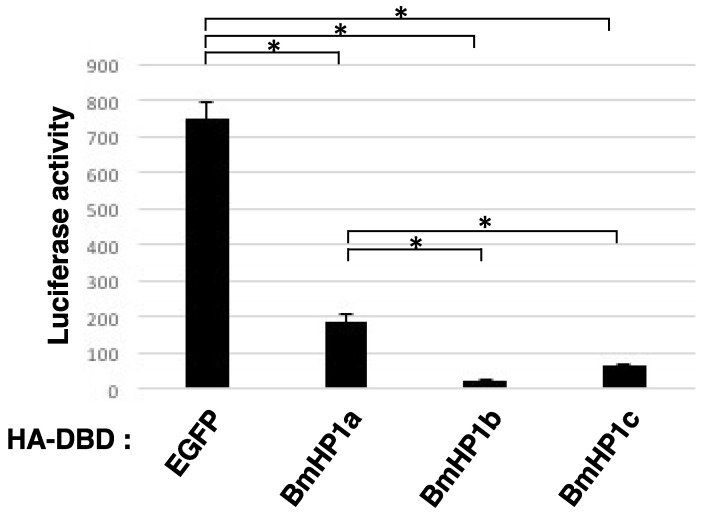
Transcription repression assay. DBD fused protein expression plasmid and luciferase reporter plasmid were co-transfected into BmN4 cells. After 5 days, the cells were collected and the luciferase activity was measured to evaluate the transcription repression activity of *BmHP1*s. Error bars = standard deviation (*n* = 3). “*” indicates a significant difference at *p* < 0.05 (Tukey test).

Subsequently, a transcription repression assay was performed to confirm whether BmHP1c has a transcription repression activity as described under the Materials and Methods. As shown in Figure 2, the recruitment of each three *BmHP1* in the promoter region drastically reduced the luciferase activity, compared to that of DBD-*EGFP*. In the transcription repression assay, there was a significant difference in each experimental group (*p* < 0.05, Tukey test), except for the comparison between BmHP1b and BmHP1c. Among these BmHP1s, BmHP1b shows the strongest transcription suppression, and BmHP1c has the second-highest transcriptional repression ability. The relatively low transcriptional repression activity of BmHP1a may reflect the functional duality of transcriptional repression in the transposon region and transcriptional activation in the euchromatin region.

### 3.3. BmHP1c Does Not Form Homo- and Heterodimers with the Other BmHP1s

**Figure 3:**
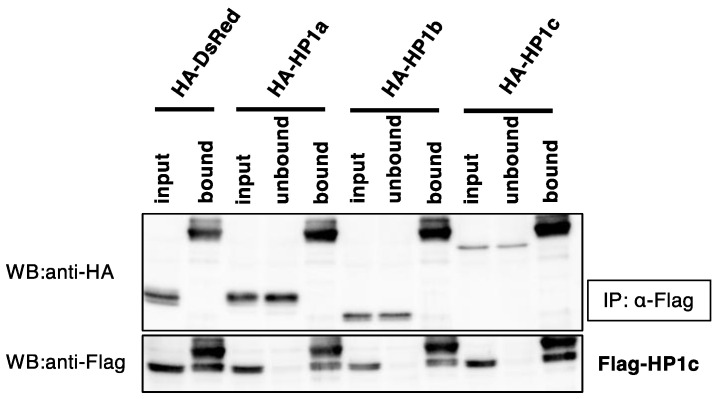
Co-immunoprecipitation of BmHP1c and *BmHP1*s. Flag-BmHP1c and HA-*BmHP1*s expression plasmids were co-transfected into BmN4 cells, and 5 days later, the cells were collected and co-immunoprecipitation was performed. Input samples are cell lysates, unbound samples are samples that did not bind to the antibody-coupled G protein beads, and bound samples are bound to antibody-coupled G protein beads.

In order to determine whether BmHP1c forms homo- and heterodimeric complexes with the other BmHP1s, co-immunoprecipitation experiments were performed as described under the Materials and Methods. When the Flag-BmHP1c and HA-BmHP1s were co-expressed in silkworm cultured cells, and Flag-BmHP1c was immunoprecipitated using anti-Flag antibody, none of HA-BmHP1s were co-immunoprecipitated with BmHP1c (Figure 3). In inverse combination, Flag-BmHP1a was co-immunoprecipitated with HA-BmHP1a and HA-BmHP1b but not with HA-BmHP1c (Figure S1).

### 3.4. Tissue-Specific Expression and Subcellular Localization of the BmHP1c in Silkworm Cultured Cell

**Figure 4:**
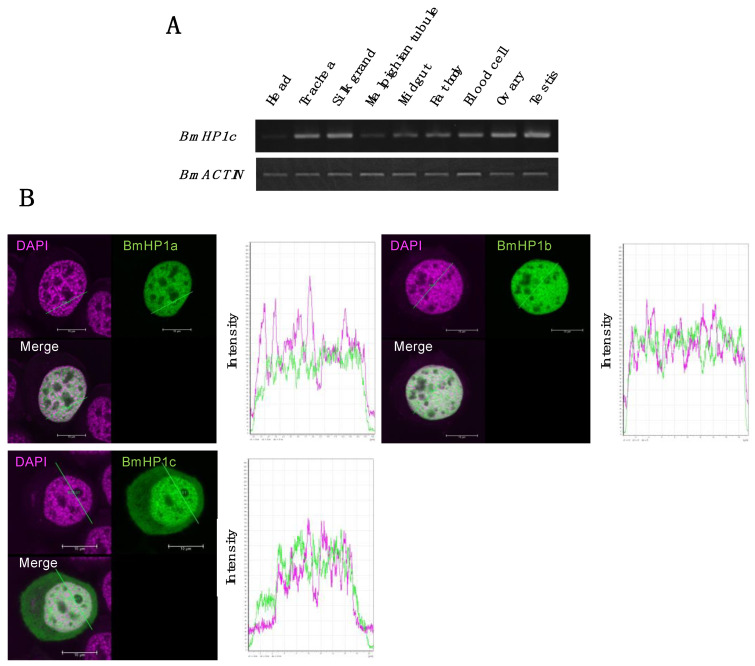
(**A**) *BmHP1c* expression analysis for each tissue. Total RNA was extracted from each silkworm tissue, cDNA was synthesized, and then PCR was performed with *BmHP1c* specific primers. *BmACTIN* was used to normalize the variability in template loading. (**B**) Localization of BmHP1s in BmN4 cultured cells. A vector expressing *mClover3* fused BmHP1s was transfected into BmN4 and observed with a confocal microscope. Left panel: photomicrograph. Right panel: The fluorescence intensity at the position indicated by the green line in the right panel.

In *Drosophila melanogaster*, five HP1 paralogs (*DmHP1a*, *DmHP1b*, *DmHP1c*, *DmHP1d* and *DmHP1e*) were identified. *DmHP1a*, *DmHP1b*, and *DmHP1c* are ubiquitously expressed, while *DmHP1d* is specifically expressed in the ovary, and *DmHP1e* are predominantly expressed in the testis [23]. To infer the function of BmHP1c in detail, the transcription profile was analyzed using nine tissues of fifth instar third-day larvae (Figure 4A). RT-PCR revealed the ubiquitous expression of *BmHP1c*, but the expression level decreased in the head and malpighian tubule in which cell proliferation and cell enlargement are slowed.

In order to identify the subcellular localization of BmHP1c in silkworm cultured cells, we performed intracellular localization analysis using the fluorescent protein *mClover3* fused BmHP1c. Confocal laser microscope observation revealed that BmHP1c exists not only in the nucleus but also in the cytoplasm during interphase (Figure 4B), unlike BmHP1a and BmHP1b localizing in the nucleus [18]. BmHP1c may have different functions from BmHP1a and BmHP1b. In addition, in ovary-derived BmN4 cells, a small amount of three BmHP1s was present in the regions strongly stained with DAPI compared with the regions weakly stained with DAPI, although none of the three BmHP1s were present in regions with extremely low DNA content (almost impossible to stain with DAPI), which may be nucleoli (Figure 4B).

### 3.5. Knockdown of BmHP1c Does Not Affect Cell Cycle Progression

**Figure 5:**
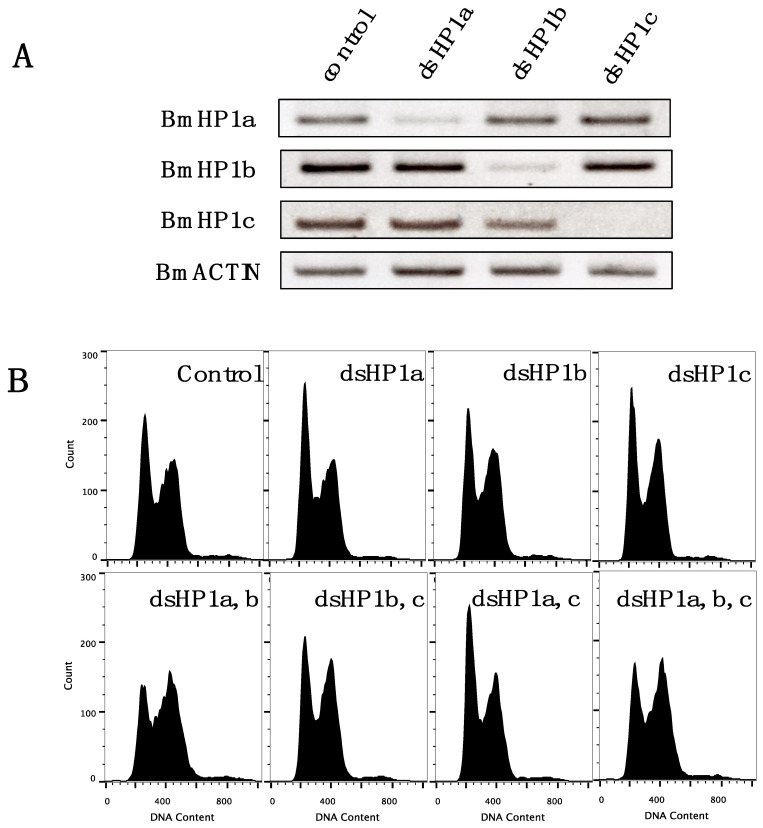
*BmHP1s* knockdown experiment. (**A**) Confirmation of *BmHP1s* knockdown efficiency by RT-PCR. *BmACTIN* was used to normalize the variability in template loading. (**B**) Flow cytometric analysis of the cell cycle in the cells inducing RNAi of *BmHP1s* for 10 days. Five days after the knockdown, the cells were subcultured on a new plate, at which time dsRNA was also added.

In order to investigate the function of BmHP1c, the knockdown experiment of *BmHP1s* was performed using the BmN4-SID1 cell line, which can induce RNAi by the addition of dsRNA to the medium [22]. As shown in Figure 5A, RT-PCR indicated that all three *BmHP1s* were successfully knocked down by soaking RNAi. Subsequently, flow cytometry measurements were performed to investigate the effects on cell cycle progression 10 days after the gene knockdown (Figure 5B). After 5 days of the treatment, no significant effect was observed even with a triple knockdown. In the 10-day treatment, the double knockdown of *BmHP1a* and *BmHP1b* arrested the cell progression in the G2/M phase, but no similar effect was observed in the double knockdown of *BmHP1a* and *BmHP1c* or *BmHP1b* and *BmHP1c*.

## 4. Discussion

In this study, we report a novel *BmHP1c* as the third *Bombyx mori HP1* gene. Compared to *Homo sapiens* and *Drosophila melanogaster*, where three and five *HP1* genes have been reported, respectively, only two *HP1* genes, *BmHP1a* and *BmHP1b*, have been reported in the silkworm [18]. BmHP1c had stronger transcriptional repressive activity than previously reported BmHP1a and weaker than BmHP1b. The reason for the weaker transcriptional repressive activity of BmHP1a compared to BmHP1b [18] may be its unique functional duality, that is, BmHP1a upregulates the transcription of some genes bound in the upstream region, while it represses the transcription of transposons in the telomere region, as in other organisms [19]. It remains the possibility that BmHP1c, which shows intermediate transcriptional repressive activity, also has this duality.

Subcellular localization analysis showed that all three *BmHP1*s were abundant in the euchromatin region, which is not well stained by DAPI. This phenomenon is similar to that observed in *Arabidopsis*, which has only one *HP1* homolog, *LHP1*. *Arabidopsis LHP1* is preferentially located in the euchromatin region and plays a role in repressing gene expression in the euchromatin region rather than in the heterochromatin region [24].

There are at least two types of heterochromatin, constitutive heterochromatin, and facultative heterochromatin, and it is generally believed that HP1 is involved in the formation of constitutive heterochromatin around telomeres and centromeres. In BmN4 cells, HP1s are poorly present in the regions stained well with DAPI, suggesting that this strongly staining region is mainly a facultative heterochromatin formed by polycomb group proteins. On the other hand, in *Drosophila*, DmHP1a and DmHP1b are present in the heterochromatin region that is well stained by DAPI [14]. Considering these results and the fact that the silkworm has a unique chromosome structure called the holocentric chromosome, there are three possibilities. (1) The pericentric region of the silkworm is also stained by DAPI but is not long enough to be observed under the microscope, (2) the pericentric region on the holocentric chromosome is structurally different from other organisms and is not stained by DAPI, or (3) another protein, not HP1, replaces the role of HP1 in the pericentric region of the silkworm.

Interestingly, BmHP1c was localized not only in the nucleus but also in the cytoplasm. Similarly, experiments using S2 cells have shown that DmHP1b and DmHP1c are present in the cytoplasm as well as the nucleus and that the C-terminal extension regions of DmHP1b and c play an important role in cytoplasmic localization [14]. The length of the C-terminal tail of DmHP1a, b, and c is 3, 85, and 96 amino acid residues, respectively [14], while those of BmHP1a, b, and c are 7, 12, and 64 amino acid residues, respectively. The cytoplasmic localization of BmHP1c may be attributed to the C-terminal extension region of BmHP1c.

It has been reported that mammalian *HP1γ* is also present in the cytoplasm [25] and that *HP1γ* translocates between the nucleus and cytoplasm during the development of bovine embryo [26]. It has been suggested that *HP1γ* may regulate gene expression by translocating from the nucleus to the cytoplasm. For example, in human papillomavirus-positive cervical cancer cells, which show abnormal localization of *HP1γ* to the cytoplasm, inhibition of *HP1γ* translocation to the cytoplasm results in decreased expression of *UBE2L3*, which is involved in p53 degradation, resulting in altered transcription of p53 target genes [27]. However, the biological significance of the presence of *HP1* in the cytoplasm is largely unknown.

In *Drosophila*, heterodimer formation between DmHP1a and DmHP1c could not be confirmed by Co-IP assay, but in vitro binding assays reported that ubiquitously expressed DmHP1a, b, and c form homo- and heterodimers in all combinations [14]. The homodimer formation of DmHP1d has also been confirmed in a two-hybrid assay [28]. On the other hand, the homodimer of BmHP1a and BmHP1b has been confirmed in the Co-IP assay in the silkworm [18], but the formation of homo- and heterodimers of BmHP1c have not been confirmed in this study. It remains possible that weak interactions are not detectable in the Co-IP assay, as in the case reported in *Drosophila*. HP1s interact through CSDs with each other [8,29,30,31] and with other transcriptional regulators [7,32,33], we constructed a phylogenetic tree of CSDs and found that BmHP1a and BmHP1b belong to the same cluster, while BmHP1c belongs to a different cluster. BmHP1c may play a unique role in transcriptional regulation through protein–protein interactions that are different from those of other silkworm HP1s.

Furthermore, BmHP1c showed a different phenotype from other BmHP1s in knockdown experiments using ovary-derived BmN4 cultured cells. That is, the double knockdown of *BmHP1a* and *BmHP1b* had a clear effect on the cell cycle, whereas the double knockdown of *BmHP1a* and *BmHP1c* and *BmHP1b* and *BmHP1c* had no effect on the cell cycle. This result suggests that BmHP1a and BmHP1b may have overlapping functions but no functional overlap with BmHP1c in the aspect of the cell cycle progression. Additionally, the fact that *BmHP1c* expression varies among tissues suggests that it may be required for tissue differentiation at the individual level, although it is not essential for cell survival itself. Genes involved in the development and morphogenesis of the nervous system have been reported as genes whose expression is altered when *DmHP1c* is knocked down [12], so we will conduct genome editing knockout experiments in individual silkworms to clarify the function of BmHP1c in different tissues.

## Figures and Tables

**Table 1:** Gene-specific primers used for semi-quantitative RT-PCR.

Name	Sequence
*BmHP1a*-RT5	GGTAAAAAAGAGAAGAAAACGGAGACCAG
*BmHP1a*-RT3	CGCCTATAATTTTCTCAGCTTTGAGTCC
*BmHP1b*-RT5	GCCGACAAGAAAAAAGAAAATGAACCAG
*BmHP1b*-RT3	TTCATGAGGAACATGAGTTCACCACTAC
*BmHP1c*-RT5	CAACACTTGGGAACCAGAAGACAACC
*BmHP1c*-RT3	CTCGCGATTCAACTGCTTCACTGTTAG
*BmActin*-RT5	GCATCATACCTTCTACAATGAGC
*BmActin*-RT3	GAGATCCACATCTGTTGGAAG

## Data Availability

The data presented in this study are available in the article or Supplementary Materials.

## Supplementary Materials

The following supporting information can be downloaded at: https://www.mdpi.com/article/10.3390/insects13070631/s1, Figure S1: Co-immunoprecipitation of BmHP1a and BmHP1s. Flag-BmHP1a and HA-BmHP1s expression plasmids were co-transfected into BmN4 cells, and 5 days later, the cells were collected and co-immunoprecipitation was performed.
