# Supplementary figures and images for: Characterization of a Novel Heterochromatin Protein 1 Homolog “HP1c” in the Silkworm, Bombyx mori
Source: Insects. 2022 Jul 14;13(7):631. doi: 10.3390/insects13070631 (PMC9316600; doi:10.3390/insects13070631)

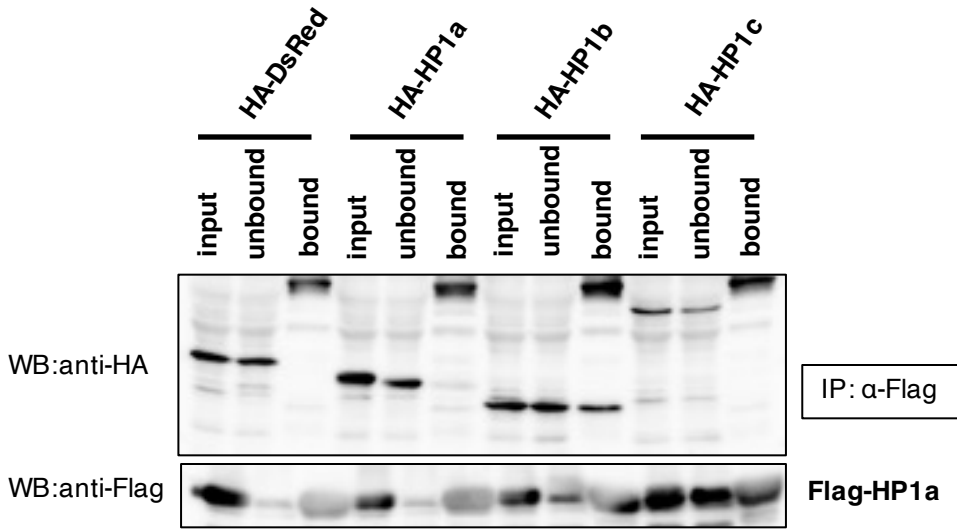

Supplement: Supplementary file 1 [file insects-13-00631-s001.zip › Figure S1.pdf]

Figure 3

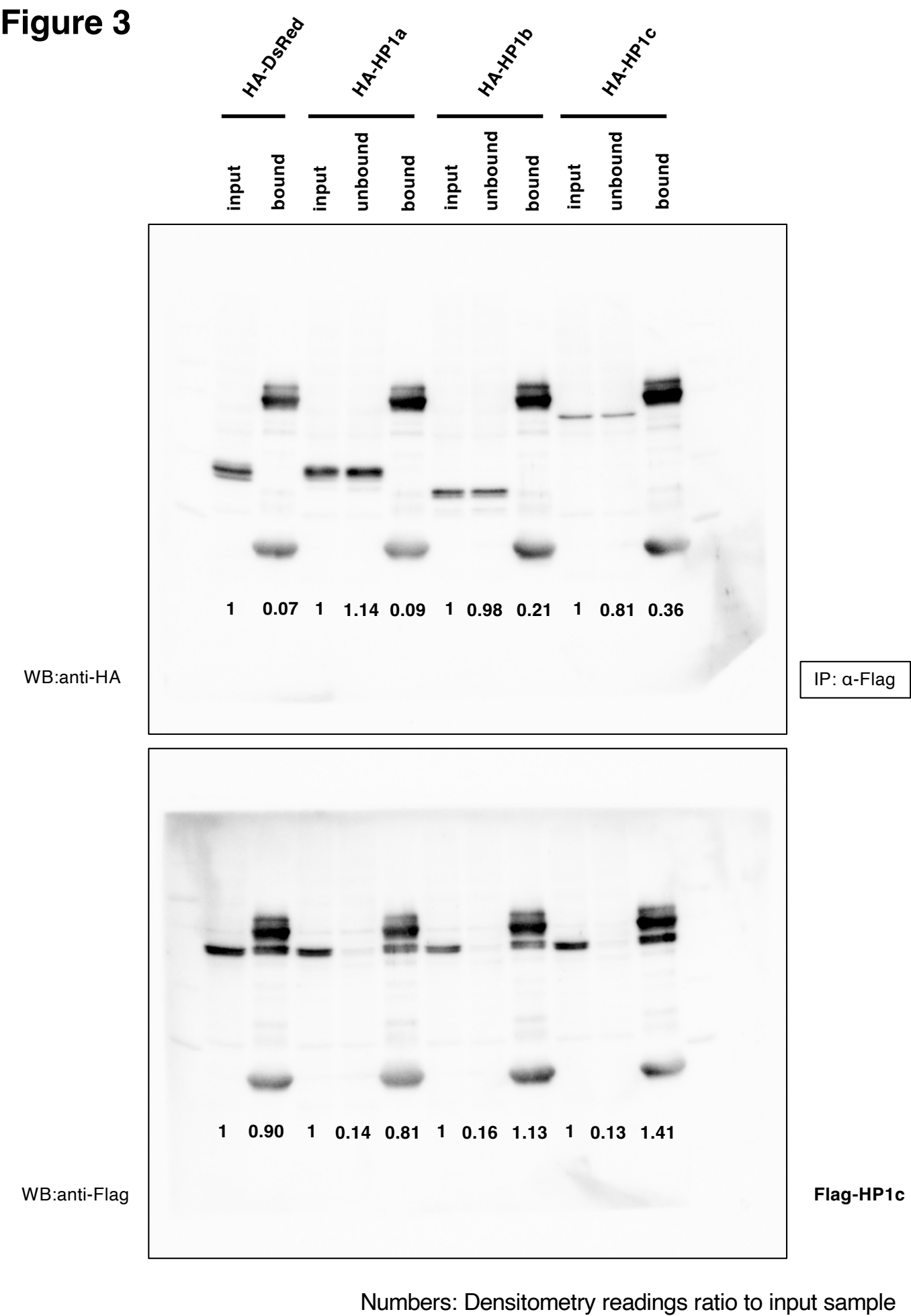

Figure S1

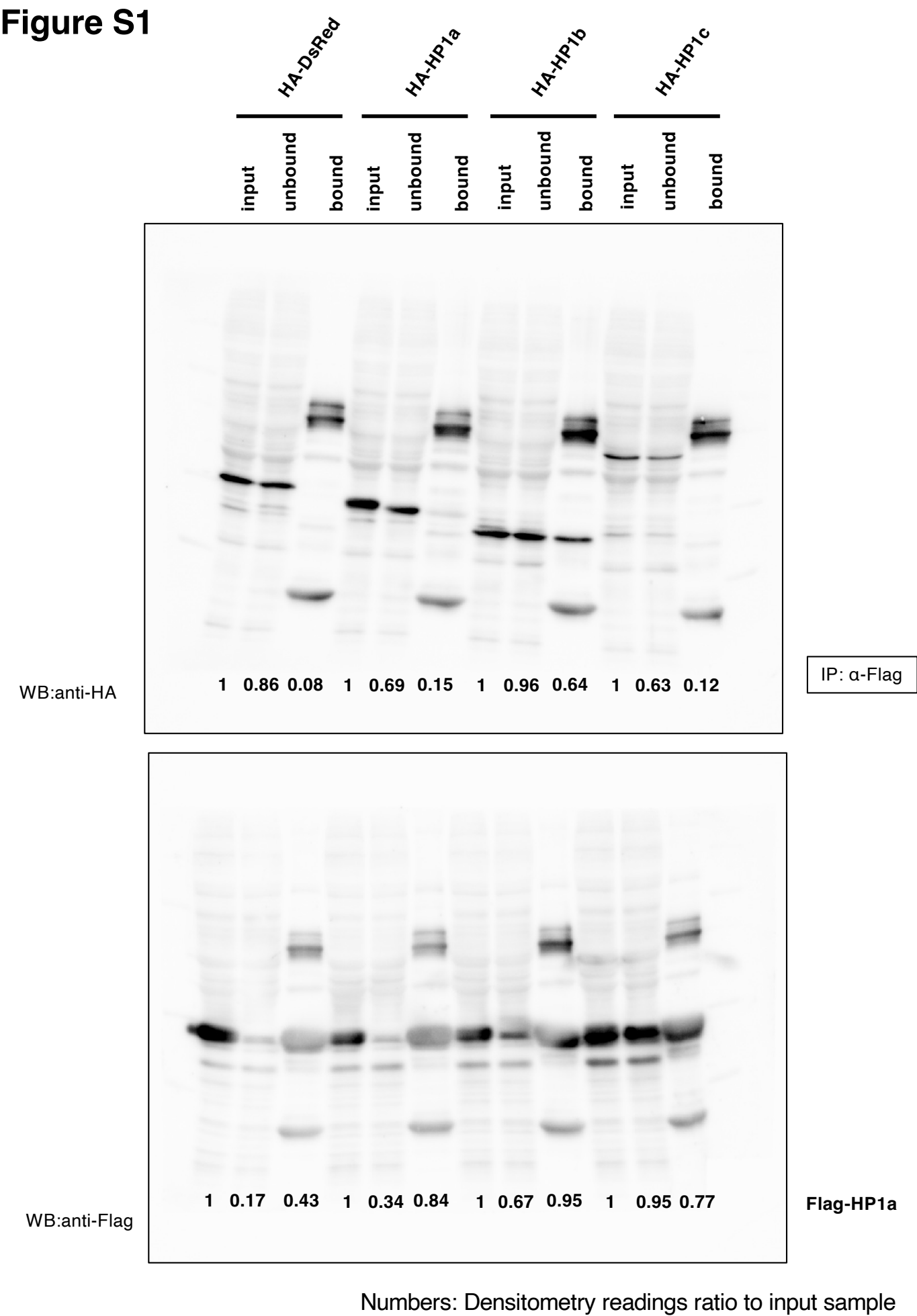

Supplement: Supplementary file 1 [file insects-13-00631-s001.zip › whole blot of Figure3-S1.pdf]
